# Control of TCF-4 Expression by VDR and Vitamin D in the Mouse Mammary Gland and Colorectal Cancer Cell Lines

**DOI:** 10.1371/journal.pone.0007872

**Published:** 2009-11-17

**Authors:** Marcy E. Beildeck, Md Islam, Salimuddin Shah, JoEllen Welsh, Stephen W. Byers

**Affiliations:** 1 Lombardi Comprehensive Cancer Center, Department of Oncology, Georgetown University School of Medicine, Washington, D. C., United States of America; 2 GenNYsis Center for Excellence in Cancer Genomics, SUNY at Albany, Rensselaer, New York, United States of America; Bauer Research Foundation, United States of America

## Abstract

**Background:**

The vitamin D receptor (VDR) pathway is important in the prevention and potentially in the treatment of many cancers. One important mechanism of VDR action is related to its interaction with the Wnt/β-catenin pathway. Agonist-bound VDR inhibits the oncogenic Wnt/β-catenin/TCF pathway by interacting directly with β-catenin and in some cells by increasing cadherin expression which, in turn, recruits β-catenin to the membrane. Here we identify TCF-4, a transcriptional regulator and β-catenin binding partner as an indirect target of the VDR pathway.

**Methodology/Principal Findings:**

In this work, we show that TCF-4 (gene name TCF7L2) is decreased in the mammary gland of the VDR knockout mouse as compared to the wild-type mouse. Furthermore, we show 1,25(OH)_2_D_3_ increases TCF-4 at the RNA and protein levels in several human colorectal cancer cell lines, the effect of which is completely dependent on the VDR. *In silico* analysis of the human and mouse TCF7L2 promoters identified several putative VDR binding elements. Although TCF7L2 promoter reporters responded to exogenous VDR, and 1,25(OH)_2_D_3_, mutation analysis and chromatin immunoprecipitation assays, showed that the increase in TCF7L2 did not require recruitment of the VDR to the identified elements and indicates that the regulation by VDR is indirect. This is further confirmed by the requirement of *de novo* protein synthesis for this up-regulation.

**Conclusions/Significance:**

Although it is generally assumed that binding of β-catenin to members of the TCF/LEF family is cancer-promoting, recent studies have indicated that TCF-4 functions instead as a transcriptional repressor that restricts breast and colorectal cancer cell growth. Consequently, we conclude that the 1,25(OH)_2_D_3_/VDR-mediated increase in TCF-4 may have a protective role in colon cancer as well as diabetes and Crohn's disease.

## Introduction

Activation of the vitamin D pathway has been associated with a decreased risk in the development and progression of many cancers (reviewed in[1]). Although epidemiologic studies are less clear regarding the association of cancer risk with serum levels of vitamin D and its metabolites, molecular biology and animal studies support a role for vitamin D in increased apoptosis and cell differentiation, and decreased cell growth. As vitamin D is a compound that is available in the diet (albeit insufficiently) as a supplement, or readily-synthesized by the body, it is an attractive candidate for chemoprevention and chemotherapy. Its clinical benefit in this capacity, however, is inhibited by dose-limiting hypercalcemia, a side-effect that develops from the primary role of vitamin D in calcium homeostasis. In an effort to utilize vitamin D in the clinic as an anti-cancer agent, efforts have been made to generate vitamin D analogs which result in reduced hypercalcemia. While these analogs have shown great promise *in vitro* and in animal models, they fall short in evoking an equivalent response in the clinic. Furthermore, a successful analog may pose a particular problem in the context of colorectal cancer, the third leading cause of cancer-related death in men and women in the US. Although the evidence for vitamin D as an anti-cancer agent in this organ is particularly strong, the GI tract is intimately involved in mediating the effects of vitamin D on calcium homeostasis. This indicates that in the colon, it may be difficult to uncouple the anti-cancer and calcium homeostatic effects of vitamin D. Although, in other studies we show that some vitamin D partial antagonists will activate the vitamin D receptor in cells which express high levels of activated β-catenin (cancer cells) but not in normal cells and may have the potential to do this [2].

Nuclear hormone receptors can influence the canonical Wnt signaling cascade by interacting with β-catenin [3]. This phenomenon may be particularly relevant in colon cancer, where 80% of cases are a harbor of APC mutations that aberrantly activate β-catenin [4], leading to accumulation of activated β-catenin in the nucleus (Reviewed in [5]). Within the nucleus, β-catenin is responsible for co-activating the transcription of genes whose promoters are occupied by members of the TCF/LEF family of transcription factors. Some of these genes such as *c-myc* [6] and *Cyclin-D1* [7], are involved in cell cycle regulation and can contribute to an oncogenic phenotype. Treatment of cells with some (but not all) nuclear hormone receptor (NHR) agonists causes an up-regulation of NHR-responsive genes while simultaneously causing a decrease in TCF/β-catenin target gene transcription. This has been attributed to competitive binding between TCF and NHRs for β-catenin [3], [8]–[11], and/or common co-activators such as p300 [2]. A second mode of inhibition of Wnt target gene transcription has been attributed to the prevention of β-catenin nuclear translocation by recruitment of cytoplasmic β-catenin to adherens junctions [9], [12], [13]. Thirdly, there is evidence that NHRs bind to TCF/LEF family members, directly, and thereby inhibit transcription of TCF/β-catenin responsive genes [14]–[18]. This is probably due to recruitment of co-repressors such as TLE (Groucho) [19], NCoR and SMRT [20].

Here we report an additional mechanism of interaction between the β-catenin pathway and the vitamin D receptor (VDR) pathway. To clarify, we will use the following nomenclature: TCF7L2 in the context of plasmids, DNA and RNA, and TCF-4 in the context of protein, only. We found that TCF-4 is differentially expressed in cells derived from DMBA-induced mouse mammary tumors from VDR wild-type and knock out mice, and the mammary glands themselves. Further exploration revealed a VDR-dependent up-regulation of TCF7L2 at the mRNA and protein levels by treatment with 1,25(OH)_2_D_3_ in CaCo2 cells. Analysis of the human and mouse TCF7L2 promoter predicted several putative vitamin D receptor binding elements proximal to the transcription start site. Although cloning of this promoter region revealed regulation by the VDR/1,25(OH)_2_D_3_, subsequent mutation analysis, chromatin immunoprecipitation and protein synthesis-inhibition experiments indicate that this effect is likely conveyed indirectly, via a VDR/1,25(OH)_2_D_3_-sensitive intermediary. Increases in TCF-4 protein levels translate to increased TopFlash activity after 24 hours of ligand treatment in CaCo2 cells. We propose a mechanism whereby this effect may inhibit oncogenic activity *in cellulo*.

## Results

### Mammary Glands from VDR Knockout Mice Have Less TCF-4 than Mammary Glands from VDR Wild-Type Mice

**Figure 1:**
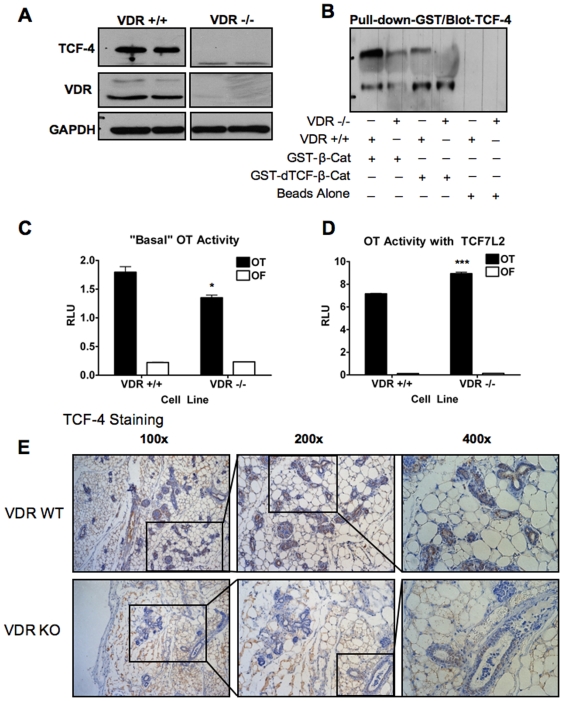
VDR145^+/+^ cells have more basal TCF-4 than VDRK240^−/−^ cells. (**A**) Western blot of whole cell lysates, in duplicate, from VDR145^+/+^ (VDR +/+) cells (lanes 1 and 2) and VDRK240^−/−^ (VDR −/−) cells (lanes 3 and 4) probed for TCF-4, VDR and GAPDH. (**B**) Pull-down of proteins in VDR +/+ and VDR −/− lysates with GST-tagged β-catenin constructs and probed for TCF-4. GST-WT-β-catenin (GST-β-Cat) is used in lanes 1 and 2. Mutated β-catenin that will not effectively bind TCF/LEF proteins (GST-dTCF-β-Cat) is used in lanes 3 and 4. Beads alone are used in lanes 5 and 6. (**C**) OT activity in VDR +/+ and VDR −/− cells transfected with *Renilla* and VP16-β-catenin. Data are normalized to *Renilla*. Error bars represent SEM. p-values represent student's t-test for significan differences between cell lines. RLU: Relative Light Units (**D**) OT activity in VDR +/+ and VDR −/− cells transfected with *Renilla*, VP16-β-catenin and with or without a TCF7L2 plasmid. Error bars represent SEM. Statistics are student's t-test for differences between transfected and control-transfected cells. *p<.05; ***p<.0005. (**E**) Mammary gland tissues from VDR WT (top panels) and VDR KO (bottom panels) mice stained for TCF-4 shown at three magnifications.

VDR145^+/+^ and VDRK240^−/−^ cell lines are derived from DMBA-induced mouse mammary tumors from wild-type (WT) and VDR knockout (KO) mice, respectively and were described previously [21]. VDR KO mice are more susceptible to carcinogen-induced mammary and colon carcinogens as well as other lesions [22], [23]. Preliminary experiments indicated that TCF-4 was more abundant in WT cells than KO cells (not shown). We performed western blot analysis for TCF-4 and confirmed that VDRK240^−/−^ cells have very low levels of TCF-4 compared to VDR145^+/+^ cells (Figure 1A). We next performed a pull-down assay in which we used two GST-tagged β-catenin fusion proteins. WT GST-tagged β-catenin binds TCF/LEFs via the armadillo repeat region, whereas GST-dTCF-β-Cat, which harbors mutations at residues 253, 312, and 435 within the armadillo region, has dramatically-reduced affinity for TCF/LEFs [24]. Consistent with the western blot data, the WT fusion protein pulled down more TCF-4 from VDR145^+/+^ lysates than from VDRK240^−/−^ lysates. The mutated fusion protein pulled-down much less TCF-4 from the VDR145^+/+^ cells and none from the VDRK240^−/−^ cells (Figure 1B).

We next used the OT/OF reporter system to assay TCF/β-catenin activity in these cells. The OT reporter contains three tandem TCF/LEF binding elements upstream of a luciferase gene and the corresponding control reporter (OF) has mutated TCF binding sites and represents background binding. Because these cells have low endogenous β-catenin activity, VP16-β-catenin was co-expressed with the reporter vectors in all samples. Consistent with their reduced levels of TCF-4, VDRK240^−/−^ cells had less β-catenin activity than VDR145^+/+^ cells (Figure 1C). This difference was significant but not large and indicates that VDRK240^−/−^ cells have other TCF family members that can compensate for TCF-4, and/or the low levels of TCF-4 that remain in these cells is sufficient for activation, at least in the context of high levels of activated β-catenin [25], [26]. Co-transfection with exogenous TCF7L2 rescued the reduced β-catenin activity in the VDRK240^−/−^ cells (Figure 1D). To determine whether this phenomenon was also happening *in vivo*, we stained normal mammary tissues from VDR WT and VDR KO mice for TCF-4. In concordance with the rest of the data in Figure 1, mammary tissues from VDR WT animals have more prominent staining compared to mammary tissues from VDR KO animals (Figure 1E). TCF-4 staining is not completely absent in VDR KO mammary glands, although it is much less frequent and less intense (Figure 1E, bottom right panel).

**Figure 2:**
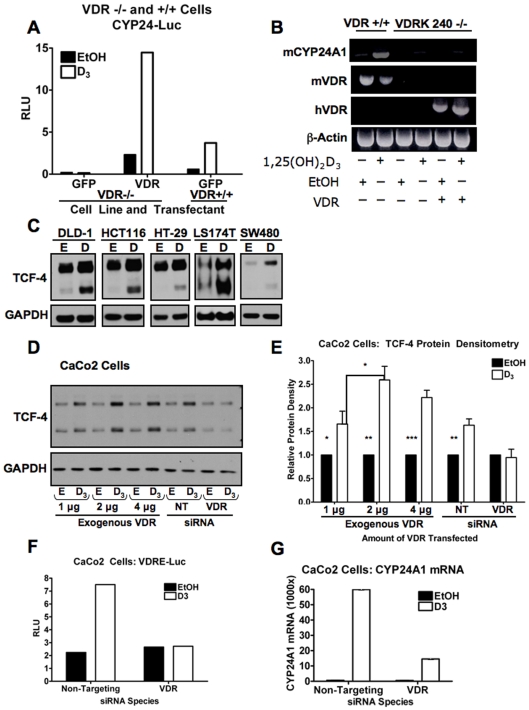
TCF-4 is increased by 1,25(OH)_2_D_3_ in a VDR-dependent manner in human colorectal cancer cells. (**A**) CYP24-luc activity in VDRK240^−/−^ (VDR −/−) and VDR145^+/+^ (VDR +/+) cells that were transfected with GFP or VDR and treated with 10^−7^ M 1,25(OH)_2_D_3_ or EtOH-control for 24 hours as indicated. RLU = Relative Light Units. (**B**) Reverse Transcriptase PCR analysis of mouse CYP24A1 (top panel), mouse and human VDR (middle panels) and β-actin (bottom panel) transcripts in response to exogenous human VDR expression and 1,25(OH)_2_D_3_ treatment as described in part A. (**C**) Various colorectal cancer cell lines were incubated for 24 hours in 10^−7^ M 1,25(OH)_2_D_3_ or EtOH, as indicated. Cellular proteins were assayed for TCF-4 expression (upper panel) and GAPDH was monitored for equal lane loading (lower panel). Both TCF-4 bands represent different isoforms of TCF-4 that have different length C-termini. (**D**) CaCo2 cells were transfected with different amounts of VDR or siRNA for 24 hours and treated with 10^−7^ M 1,25(OH)_2_D_3_ or EtOH for a subsequent 24 hours, as indicated, and probed for TCF-4. NT: Non-targeting. (**E**) Densitometric analysis of three western blots as indicated in part A. Data were plotted relative to each EtOH-treated control. p-values were generated by one-tailed t-test: * p<.05; ** p<.005; *** p<.0005 (**F**) CaCo2 cells were transfected for 24 hours with VDRE-luc, *Renilla* and siRNA and treated for a subsequent 24 hours with 10^−7^ M 1,25(OH)_2_D_3_ as indicated. RLU: Relative Light Units. (**G**) CaCo2 cells were transfected with siRNA for 24 hours and treated for a subsequent 24 hours with 10^−7^ M 1,25(OH)_2_D_3_ or EtOH as indicated. CYP24A1 transcripts were assayed by qPCR. Data are normalized to GAPDH expression and plotted relative to each EtOH control.

CYP24A1 is the best-characterized, downstream target of the vitamin D pathway and its mRNA is exquisitely sensitive to VDR agonists. CYP24A1 is involved in the metabolism of active vitamin D compounds into inactive metabolites. Activity of the CYP24A1 reporter was absent and not affected by 1,25(OH)_2_D_3_ in VDRK240^−/−^ cells but was restored upon transfection of VDR (Figure 2A). Collectively, these data show that cells that are null for the VDR have lower basal levels of TCF-4 and that this diminishes β-catenin activity in our VDR-null mammary model.

### VDR/1,25(OH)_2_D_3_ Regulates TCF7L2 mRNA and Protein in Colorectal Cancer Cell Lines

We next transfected VDRK240^−/−^ cells with human VDR and found that TCF-4 protein and TCF7L2 mRNA were unaffected by VDR or 1,25(OH)_2_D_3_ (Supplementary Figure S1A and S1B). Remarkably, even though the activity of the CYP24A1 reporter was sensitive to exogenous VDR and 1,25(OH)_2_D_3_ in these cells (Figure 2A), like TCF7L2, *endogenous* CYP24A1 mRNA was not, despite transfection optimization that allowed us to achieve 60% efficiency (Figure 2B). These data indicate that VDRK240^−/−^ cells have long-lived alterations in the ability of endogenous target genes to respond to the transient restoration of VDR. As exogenously expressed promoters are sensitive, this is likely a result of changes in chromatin organization which cannot be reversed by short term expression of VDR and treatment with 1,25(OH)_2_D_3_.

**Figure 3:**
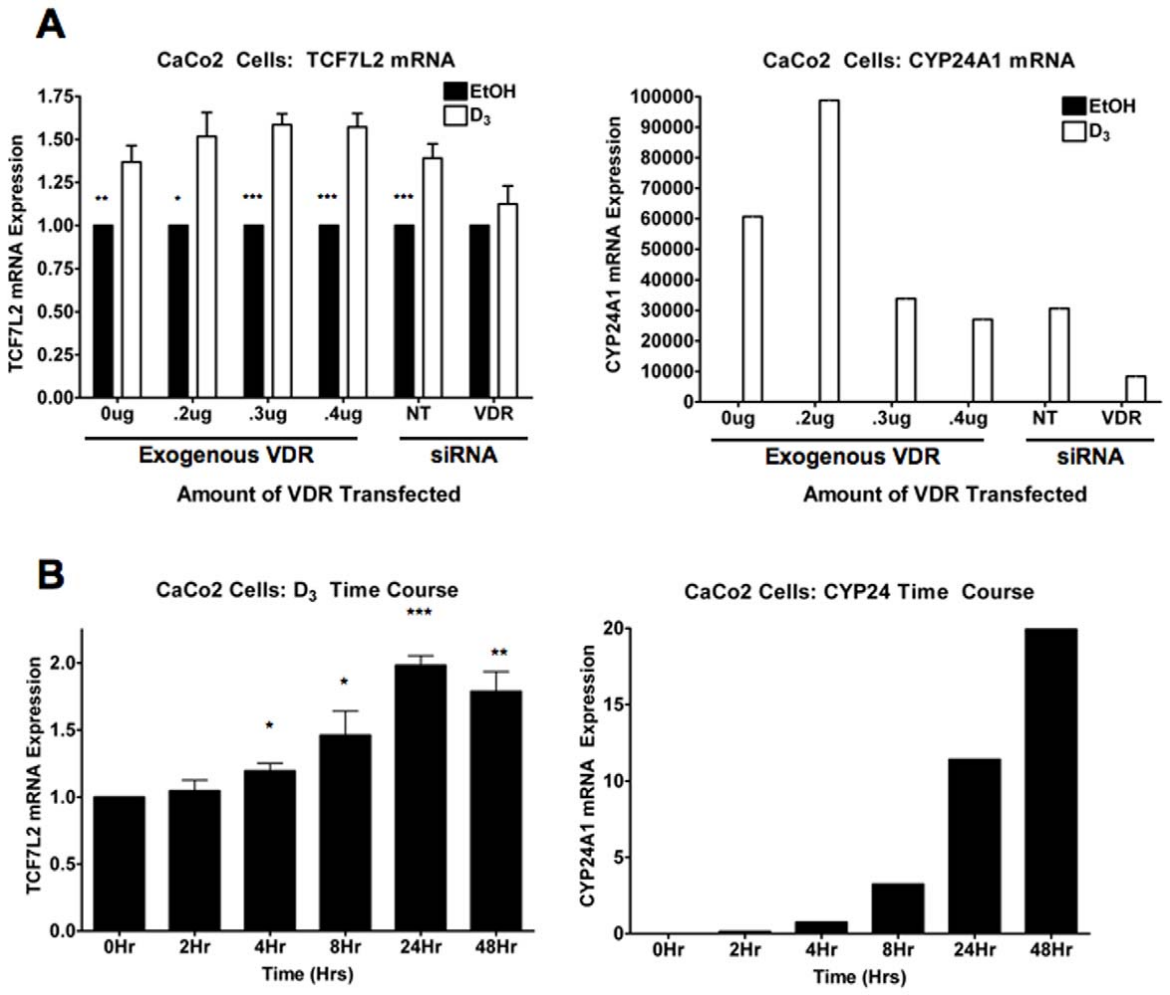
Characterization of TCF7L2 up-regulation in response to 1,25(OH)_2_D_3_. (**A**) qPCR analysis of CaCo2 cells transfected and treated as described in Figure 2D and 2E. TCF7L2 (left panel) and CYP24A1 (right panel) transcripts. Data are normalized to GAPDH and plotted relative to each EtOH-treated control. p-values are derived from student's t-test comparison between EtOH and D_3_ controls: * p<.05; **; p<.005; *** p<.0005 (**B**) CaCo2 cells were treated at different time points with 10^−7^ M 1,25(OH)_2_D_3_ up to 48 hours before collection. mRNA for TCF7L2 (left panel) and CYP24A1 (right panel) was assayed by qPCR. Data were normalized to GAPDH and plotted as fold change relative to the 0 Hr timepoint. p-values were derived from student's t-test as compared to 0 Hr timepoint: * p<.05; **; p<.005; *** p<.0005.

Consequently, to further determine the effects of VDR and its classical ligand on TCF-4 expression, we assayed several colorectal cancer cell lines for changes in TCF-4 expression in response to 1,25(OH)_2_D_3_ (Figure 2C, Supplementary Figure S2). Although all of these cells increased expression of TCF4 in response to vitamin D we chose to use the human colorectal adenocarcinoma cell line, CaCo2 for several reasons. These cells are well-studied in the context of vitamin D signaling and their growth is inhibited by 1,25(OH)_2_D_3_ [27]. Furthermore, they are a unique system that mimics normal gut epithelium as once they become confluent they differentiate in culture [28]. These cells also express VDR, but at lower (i.e. normal) levels with respect to several other colon cancer cell lines [29], [30]. Treatment of confluent CaCo2 cells with 10^−7^ M 1,25(OH)_2_D_3_ with or without transfection of VDR lead to a robust and statistically significant increase in TCF-4 protein (Figure 2D and E). The effects of 1,25(OH)_2_D_3_ are modestly potentiated by exogenous VDR and this is likely an underestimate as only 50–60% of the cells are transfected. More importantly, the increase in TCF-4 induced by 1,25(OH)_2_D_3_ is absolutely dependent on the VDR, as its knockdown inhibits not only the effects of 1,25(OH)_2_D_3_ on the activity of a VDRE promoter reporter and on CYP24A1 mRNA induction, but also on TCF-4-induction (Figure 2D–G). In contrast to VDRK240^−/−^ cells (Supplementary Figure S1B), TCF7L2 and CYP24A1 mRNAs were increased by 1,25(OH)_2_D_3_ in CaCo2 cells (Figure 3A). Both TCF7L2 and CYP24A1 levels were elevated after 4 hours with TCF7L2 reaching a maximum induction at 24 hours (Figure 3B). This regulation is also subject to cell density, as statistically significant differences in TCF7L2 mRNA are only seen at higher density (Supplementary Figure S3). This may be analogous to the modestly potentiated effects of exogenous VDR on 1,25(OH)_2_D_3_-mediated increase of TCF-4 (Figure 2E), as CaCo2 cells are known to up-regulate VDR upon confluence/differentiation [31].

### VDR/1,25(OH)_2_D_3_ Regulates the Activity of the TCF7L2 Promoter in Mouse Mammary and Human Colorectal Cancer Cells

**Figure 4:**
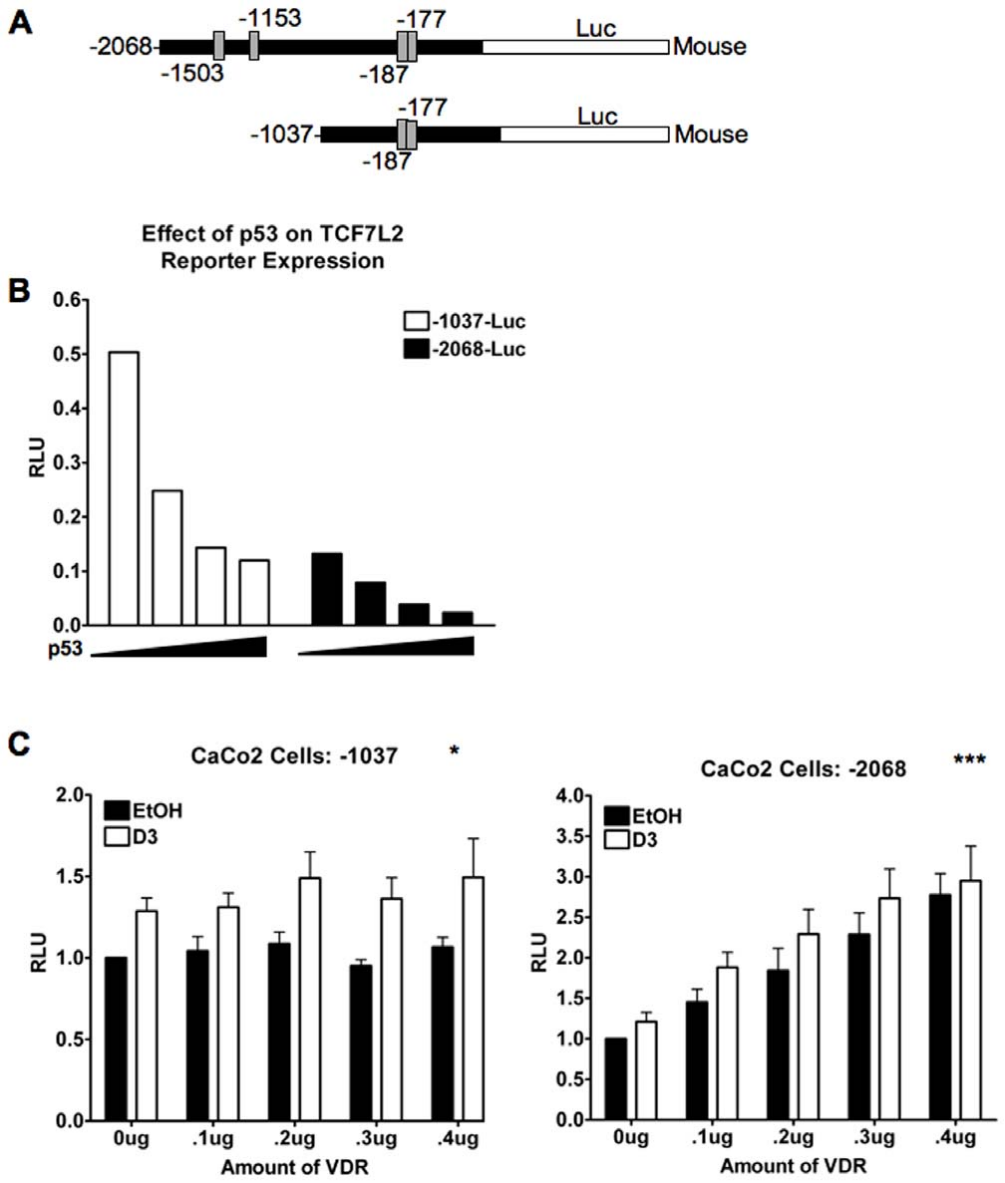
Mouse TCF7L2 promoter reporter constructs respond to VDR or 1,25(OH)_2_D_3_. (**A**) Two mouse TCF7L2 promoter constructs were cloned upstream of a luciferase reporter. The four putative VDRE locations relative to the translation start site are denoted, and all VDREs are encoded on the non-coding strand. The −177 and −187 VDREs are overlapping and share a half-site between them. The −1037 construct (-1037-luc) contains the region from −1 to −1037 relative to the translation start site and possesses the −177/−187 VDRE, only, while the −2068 reporter (-2068-luc) contains the region between −96 and −2068 relative to the translation start site of the mouse TCF7L2 promoter, and possesses all four putative VDREs. (**B**) CaCo2 cells were transfected for 24 hours with *Renilla* and reporters as indicated as well as different amounts of p53. Data are normalized to *Renilla* and to empty reporter expression. RLU = Relative Light Units. (**C**) -1038-Luc or empty vector constructs were transfected into CaCo2 cells with *Renilla* and different amounts of VDR, as indicated. 24 hours after transfection, cells were treated for an additional 24 hours with 10^−7^ M 1,25(OH)_2_D_3_ or EtOH vehicle control. Error bars represent SEM. Statistics were derived from two-way ANOVA for differences between EtOH- and 1,25(OH)_2_D_3_-treated samples: * p<.05; **; p<.005; *** p<.0005. (**D**) CaCo2 cells were transfected, treated and analyzed as in part C using -2068-luc instead of -1038-luc. Statistics were generated by two-way ANOVA for significant differences between the amount of VDR transfected: *** p<.0005. Error bars represent SEM. RLU: Relative Light Units.

Since TCF7L2 is regulated by 1,25(OH)_2_D_3_ at the mRNA level in CaCo2 cells, we scanned the mouse and human TCF7L2 genes approximately 4000 base-pairs upstream of the transcription start site for the presence of half-sites that conform to the VDRE consensus RGKTSA (R = A or G, K = G or T; S = G or C), or half-sites that deviate slightly from the consensus but that have been described as functional VDRE half-sites in the literature. We identified several putative VDREs encoded on the TCF7L2 promoter of both the human and mouse gene (Supplemental Table S1 [32]–[41]). Since the mouse and the human TCF7L2 promoter share more than 80% sequence identity in the first ∼2000 base pairs, we cloned two portions of the mouse promoter into a luciferase construct (Figure 4A). One reporter, -1037-luc, contains the region between +522 and −515 basepairs relative to the transcription start site and contains the 5'UTR and the predicted TATA box at −25 basepairs. The -2068-luc reporter spans the region between +430 and −1542 relative to the transcriptional start site. The plasmid names and subsequently-mentioned VDREs refer to the distance from the start codon and not the transcription start site since database transcription start sites vary. The half-sites of the −187/−177 DR4 elements, (two hexameric half-sites arranged as direct repeats spaced by four nucleotides) share one half-site between them.

Reporter -1037-luc contains the first putative, compound VDRE only, while the -2068-luc reporter contains all four VDREs. p53 decreases the activity of a human TCF7L2 reporter and we found that p53 also inhibited the activity of both -2068-luc and -1037-luc mouse reporters [42], [43] (Figure 4B). Furthermore, basal activity of the -2068-luc reporter was markedly less than the -1037-luc reporter indicating the presence of a strong repressor element in this region (Figure 4B). In CaCo2 cells, the -1038-luc construct responds modestly, but significantly, to the addition of ligand but the effect was not potentiated by exogenous VDR (Figure 4C, left panel). In contrast, -2068-luc responds strongly to exogenous VDR but not to treatment with 1,25(OH)_2_D_3_ in CaCo2 and VDRK240^−/−^ cells (Figure 4C, right panel and Supplementary Figure S4A, respectively). These data indicate that VDR and treatment with 1,25(OH)_2_D_3_ influences the activity of the TCF7L2 promoter but they do not demonstrate that the effect is direct.

### Putative VDREs within the Mouse TCF7L2 Promoter Are Not Important for Regulation by VDR in Mouse Mammary and Human Colorectal Cancer Cells

**Figure 5:**
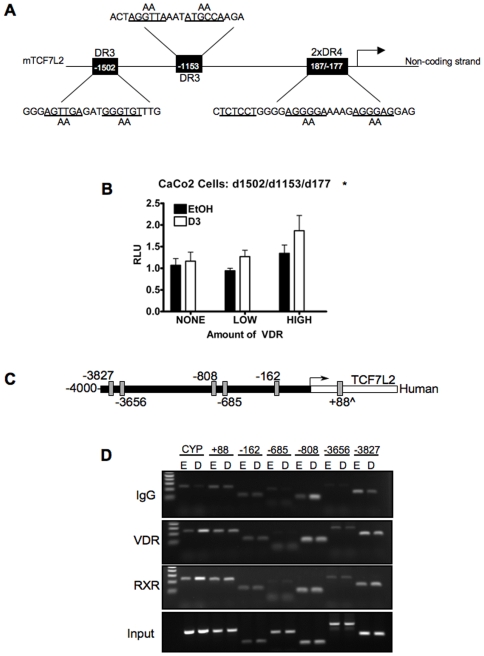
Putative VDREs within the TCF7L2 Promoter are not Responsible for 1,25(OH)_2_D_3_-VDR-mediated increase of TCF7L2. (**A**) Graphical representation of the four putative VDREs within the first ∼1500 base-pairs of the mouse TCF7L2 promoter and their sequences. DR3: direct repeat interspersed by three nucleotides. 2xDR4: two, overlapping direct repeats interspersed by four nucleotides. Although the putative VDREs are encoded on the non-coding strand, their sequences are written in reverse-complement such that the VDREs can be identified as conforming to the RGKTSA consensus. (**B**) -2068-luc construct containing all three sets of half-site mutations (d1502/d1153/d177) was transfected into CaCo2 cells and treated with ligand as described in Figure 4C with only 3 concentrations of VDR (low, medium and high). Error bars represent SEM. Statistics represent analysis using two-way ANOVA: *p<.05. RLU-Relative Light Units. (**C**) Depiction of the six putative VDREs identified within 4000 base-pairs upstream of the transcription start site of the human TCF7L2 promoter region and are named relative to their distance from the translation start site (+ being downstream, and–being upstream). All regions except +88 (marked with ^) are encoded on the non-coding strand. (**D**) Chromatin immunoprecipitation of VDR and RXR-bound DNA fragments from CaCo2 cells treated for 4 hours with 10^−7^ M 1,25(OH)_2_D_3_ (D-even-numbered lanes) or EtOH (E-odd-numbered lanes). The regions that amplify VDRE-containing regions are denoted along the top of the lanes. The CYP product amplifies a region of the human CYP24A1 promoter that is known to bind VDR and RXR in the presence of ligand and serves as a positive control. IgG represents non-specific amplification. Input demonstrates equivalent material used in each sample.

To test the hypothesis that the putative VDREs were important in conveying the response of the TCF7L2 promoter to the VDR, the third and fourth nucleotides within each half-site were mutated to double alanine residues (Figure 5A). The 5′ half-site of -187 was not mutated since the 3′half-site if this VDRE is shared with −178 VDRE, and was mutated in that construct. These mutations alter the half-sites such that they do not conform to the VDRE consensus sequence, and should not bind VDR. Mutations were generated such that each VDRE (containing two half-sites, each) was mutated in all seven possible combinations of single mutations, double mutations, and one triple mutation. Transfection of these constructs revealed that they retained responsiveness to exogenous VDR in CaCo2 cells and VDRK240^−/−^ (Figure 5B and Supplementary Figure S4B, respectively). We next performed chromatin immunoprecipitation (ChIP) assays to test the recruitment of the VDR to the six putative VDREs identified within the human TCF7L2 promoter (Figure 5C). CaCo2 cells were seeded at high density and treated for 4 hours with 10^−7^ M 1,25(OH)_2_D_3_. Both the Retinoid X Receptor (RXR) and the VDR were recruited to the published VDREs on the CYP24A1 promoter (Figure 5D, lanes 1 and 2), however, neither NHR was recruited to any of the putative VDREs in the human TCF7L2 promoter region. These data suggest that the increase in TCF7L2 expression is not due to recruitment of the VDR to these putative VDREs in the mouse and human TCF7L2 promoters. This could mean that either the VDR is being recruited to other regions of the genome and controlling the transcription of TCF7L2 directly, or the VDR is regulating this phenomenon through a 1,25(OH)_2_D_3_-sensitive intermediary.

### Regulation of TCF7L2 by VDR/1,25(OH)_2_D_3_ Is Likely Mediated by an Indirect Mechanism in Colorectal Cancer Cells

**Figure 6:**
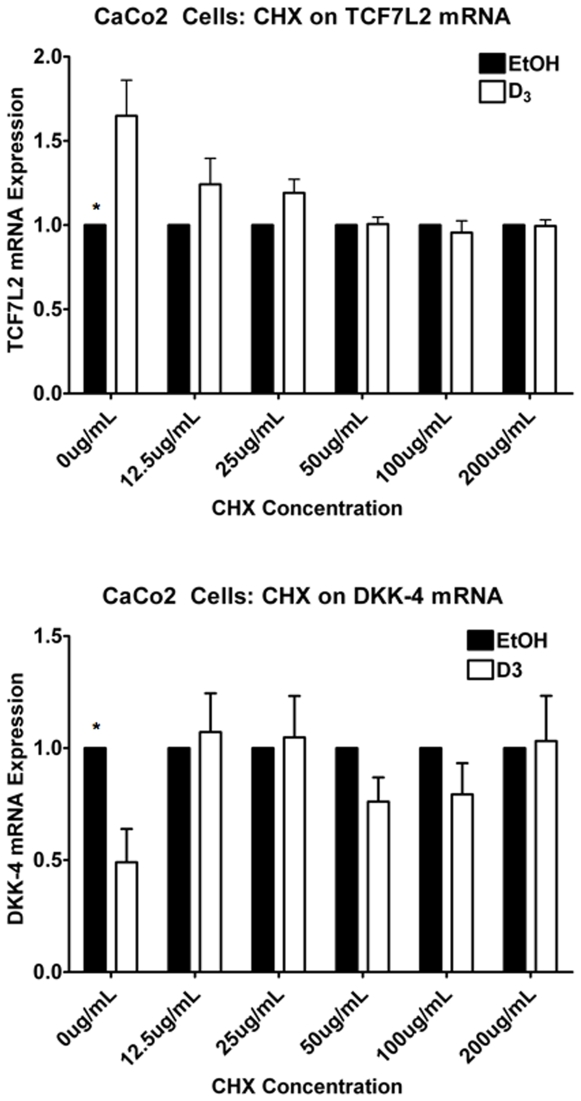
Cycloheximide treatment abolishes 1,25(OH)_2_D_3_-induced TCF7L2 mRNA induction. CaCo2 cells were pre-treated for 30 minutes with different concentrations of the protein synthesis inhibitor Cycloheximide (CHX) before addition of 10^−7^ M 1,25(OH)_2_D_3_ or EtOH for 24 hours, as indicated. Analysis of mRNA abundance of TCF7L2 (top panel) and DKK4 (bottom panel) was assayed by qPCR. Error bars represent SEM. * p<.05 via student's t-test for significant differences between D_3_ versus EtOH treated cells.

To test the latter hypothesis, we employed the translation inhibitor, cycloheximide (CHX). CaCo2 cells were pre-treated with different amounts of CHX for 30 minutes and then treated with 10^−7^ M 1,25(OH)_2_D_3_ for 24 hours. In CaCo2 cells, at concentrations as low as 12.5 µg/mL, CHX was able to significantly block induction of TCF7L2 mRNA by1,25(OH)_2_D_3_, as is DKK-4, another indirect vitamin D target that is negatively-regulated (Figure 6) [44]. These data indicate the requirement for *de novo* protein synthesis for regulation by 1,25(OH_)2_D_3._ The CYP24A1 mRNA induction by 1,25(OH)_2_D_3_ is also markedly inhibited by CHX, but is still induced ∼20 fold by 1,25(OH)_2_D_3_ at the highest concentration of CHX (Supplementary Figure S5), a phenomenon that has been shown for another direct vitamin D target gene, E-cadherin [9]. The regulation of CYP24A1 is so dramatic that it probably requires the continued synthesis of co-activator proteins to support an induction of this magnitude. Taken together, these data imply that the regulation of TCF7L2 by VDR/1,25(OH)_2_D_3_ is indirect.

### 1,25(OH)_2_D_3_ Increases TCF Reporter Activity in Colorectal Cancer Cells

**Figure 7:**
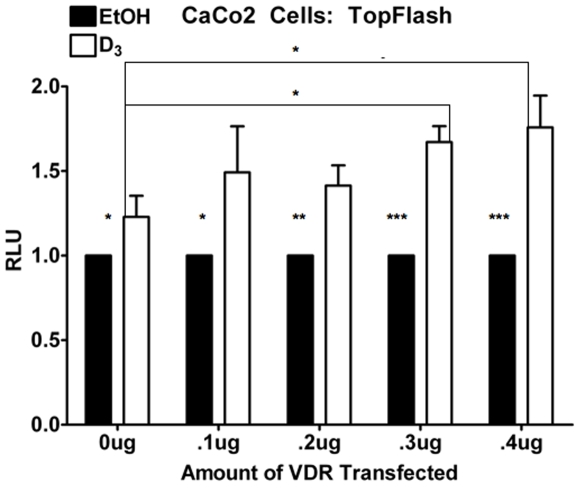
CaCo2 cells up-regulate TopFlash activity in response to 24-hour treatment with 1,25(OH)_2_D_3_. CaCo2 cells were transfected for 24 hours with TopFlash, *Renilla* and different amounts of VDR and treated for a subsequent 24 hours with 10^−7^ M 1,25(OH)_2_D_3_ or EtOH. Luciferase data were normalized to *Renilla* and FopFlash and plotted relative to each EtOH-treated control sample. Error bars represent SEM. p-values reported are from student's t-test. RLU: Relative Light Units.

While the regulation of TCF7L2 is important for many pathologic outcomes, we wanted to look at it in the context of cancer. We chose to look at the effects of VDR-mediated TCF-4 up-regulation via the classical TopFlash reporter system that has been previously described (TopFlash, unlike OT, utilizes a minimum *c-fos* promoter). CaCo2 cells have a somatic APC mutation that allows β-catenin to accumulate and translocate to the nucleus. In the case of over-abundant, active β-catenin we can expect an increase in TCF7L2 expression to result in increased TopFlash activity. Indeed, we repeated the same VDR titration experiments as described in the TCF7L2 promoter reporter experiments (Figure 4C), using TopFlash (normalized to the control plasmid, FopFlash) instead, and observed a consistent, significant increase in TopFlash activity with 24 hours of 10^−7^ M 1,25(OH)_2_D_3_ in CaCo2 cells (Figure 7) that, like the protein data (Figure 2D and 2E) is potentiated by the VDR. These data support a role for VDR-dependent, 1,25(OH)_2_D_3_-mediated increase of TCF-4 in mouse mammary and human colorectal tumor cells, the effects of which may differentially regulate β-catenin activity *in vivo*.

## Discussion

Vitamin D has shown great promise as an anti-cancer agent in both molecular and animal studies, as well as some epidemiological studies (Reviewed in [45]). In an effort to uncouple the anti-cancer effects of vitamin D from the calcemic effects that limit its usefulness in the clinic, many labs are dissecting its downstream activities. Several molecular mechanisms that contribute to the potential therapeutic action of vitamin D involve interactions with the β-catenin pathway. A direct interaction between β-catenin and a NHR, in this case, retinoic acid receptor was first described in 1999 [3]. Since then, many studies have found similar interactions for androgen receptor [8], [10], [11], peroxisome proliferator activated receptor [18], and VDR [2], [9]. These studies show that the NHR and TCF/LEF family members compete for binding to β-catenin and/or common co-activators, such as p300. In the presence of ligand, β-catenin and/or p300 favors binding to the NHR, causing a synergistic up-regulation (in conjunction with NHR ligand) of NHR-regulated genes, as well as a simultaneous down-regulation of TCF/β-catenin responsive genes. Cadherins have also been described as downstream targets of NHR pathways [9], [12], [13]. Cadherins can prevent nuclear localization of β-catenin by recruiting it from the cytoplasm to adherens junctions in the membrane, causing a putative, latent inhibition of TCF/β-catenin activity as well as a return to ‘basal’ levels of ligand-activation of NHR-responsive genes. Yet a third mechanism involves the binding of NHRs to TCF/LEF family members [14]–[18]. Many of these assays support TCF/LEF family members in their role as transcriptional repressors, likely by recruitment of other co-repressors such as TLE [19], and NCoR and SMRT [20]. Furthermore, it has been shown that once TCF/LEFs are bound by co-represssors, they are not easily displaced by activated β-catenin [17], [20].

The present study describes another mode of crosstalk between the VDR and β-catenin pathways by demonstrating that TCF7L2/TCF-4, itself, is regulated by VDR/1,25(OH)_2_D_3_ in human colorectal cancer cell lines and likely in the mammary gland as well. This induction is unique to TCF-4, as other TCF/LEF family members are not up-regulated (Supplementary Figure S6). Interestingly, LEF-1 is decreased upon treatment with ligand, an effect that may demonstrate opposing functions for LEF-1 and TCF-4 in the colon. A similar phenomenon occurs in the differentiation of keratinocytes [46]. In this case one TCF family member (TCF-3) inhibits keratinocyte differentiation and another (LEF-1) stimulates it.

Surprisingly, considering the central role of TCF7L2 in stem cell biology, cancer, and diabetes, very little is known of its regulation [43], [47]. Our initial efforts to use cells null for the VDR were hindered by the unexpected inability of these cells to respond to exogenous 1,25(OH)_2_D_3_/VDR by regulating endogenous genes. As such, we used CaCo2 cells, a cell line that is well characterized in the context of vitamin D research. These cells increase TCF7L2 mRNA and TCF-4 protein in response to 1,25(OH)_2_D_3_, an effect that is dependent upon the VDR and density. Since VDR is a transcription factor, we searched for putative VDREs within the promoters of both the human and mouse gene and although we identified numerous binding elements, none were important in the regulation of TCF7L2 by VDR/1,25(OH)_2_D_3_. Inhibition of protein synthesis abolishes 1,25(OH_)2_D_3_-mediated induction of TCF7L2 mRNA, indicating that protein synthesis is required for this effect. Although this implies that the VDR/1,25(OH)_2_D_3_ is directly regulating a modulator of TCF7L2 expression, we cannot completely preclude a direct mechanism of activation, given that the VDR may still be binding to unidentified VDREs within the TCF7L2 promoter region or even in enhancer elements far upstream or within intronic sequences [37], [48]–[50]. Although the induction of CYP24A1 mRNA is also limited by the inhibition of protein synthesis, it likely requires continued synthesis of co-activators to achieve the sheer magnitude of response to ligand. Furthermore, treatment with 1,25(OH)_2_D_3_ resulted in a 20–100 fold induction of CYP24A1 at CHX concentrations that completely block TCF7L2 induction. Thus, in combination with the reporter data, and the ChIP data, we believe that the mechanism of regulation is very likely to be indirect. Given the modest increase in TCF7L2 mRNA and the rather more robust increase in TCF-4 protein, we consider that a micro RNA (miRNA) phenomenon may be contributing, in tandem with mRNA induction via a 1,25(OH)_2_D_3_-sensitive intermediary, to the increase in TCF-4 protein. Indeed, 1,25(OH)_2_D_3_ has recently been shown to regulate the expression of some miRNAs [51].

The impact of increased TCF-4 was of particular interest in the context of CaCo2 cells, since they harbor a somatic APC mutation that allows abundant, activated β-catenin to accumulate in the nucleus. Treatment of CaCo-2 cells for 24 hours with ligand caused a modest but consistent increase in TopFlash activity which is likely due to the increase in TCF-4 protein. We propose a model whereby in the context of normal cells, or in cancers that do not have abundant activated β-catenin, an up-regulation of TCF-4 would enhance repression of TCF/β-catenin responsive genes in conjunction with the other 1,25(OH)_2_D_3_-mediated β-catenin crosstalk previously mentioned. Although β-catenin is typically associated with a cancer promoting phenotype, there are some instances where β-catenin has anti-cancer properties. Sulindac and butyrate, both of which are ascribed anti-cancer properties in the colon [52], [53] also stimulate β-catenin activity. Butyrate facilitates induction of apoptosis in a number of colon carcinoma cell lines, an effect that is thought to be partially mediated by β-catenin activity [54]. Interestingly, CaCo2 cells were one of the only cell lines that did not respond to butyrate by up-regulating TCF/β-catenin activity, however, these cells, unlike the others assayed, do not undergo apoptosis following butyrate treatment; rather they undergo differentiation [54]. CaCo2 cells do, however, undergo apoptosis in response to treatment with vitamin D agonists [55]. Thus, the short-lived up-regulation of TCF-4 by 1,25(OH)_2_D_3_ in CaCo2 cells that we report, here, may facilitate 1,25(OH)_2_D_3_-induced apoptosis in cells with abundant β-catenin, that is analogous to butyrate in other colon cancer cell lines. A unique follow up to the butyrate-induced activation of TCF/β-catenin activity showed that butyrate, also induced initiation of *c-myc* transcription in SW837 cells, presumably as a direct result of increased TCF/β-catenin activity. The elongation of this transcript, however, was halted by treatment with butyrate, such that overall *c-myc* transcripts were reduced [56]. These data indicate that β-catenin activity may be required for some anti-cancer effects, and that the pro-cancer effects may be mitigated by a simultaneous transcription-blockage mechanism. Furthermore, in colorectal cancer cells that have overactive β-catenin, TCF-4 is known to be growth inhibitory, as RNAi-mediated disruption of its expression facilitates β-catenin activity and cell growth in both DLD-1 cells (APC mutation) and HCT116 cells (activating β-catenin mutation) [57]. Recent data also show TCF-4 is lost in human breast cancers and abundant in the surrounding normal tissue, indicating that TCF-4 is a tumor suppressor in this tissue [58]. These data further support the idea that VDR and 1,25(OH)_2_D_3_-mediated up-regulation of TCF-4 has an anti-cancer role in the colon and mammary gland.

It is of particular interest to determine the downstream mediator of 1,25(OH)_2_D_3_-induced up-regulation of TCF-4. Understanding the regulation of TCF7L2 may have implications other than in the context of cancer research. For example the TCF7L2 locus shows remarkable linkage disequilibrium with type-2 diabetes (Reviewed in [59]). It is currently not understood what the risk alleles convey about the TCF-4 protein, and an obvious role for the protein in this disease is not clearly defined. 1,25(OH)_2_D_3_ has also been implicated in a protective role against type 1 and type 2 diabetes [60]–[62], and the regulation of TCF-4 we show here may play a role in disease establishment, progression, and treatment. Furthermore, lack of TCF-4 has been associated with Ileal Crohn's disease, due to downstream regulation of proteins involved in the response to bacteria [63]. Crohn's is not only a debilitating disease, but it is also believed to be a precursor to GI cancer (Reviewed in [64]). The discovery of additional regulators of TCF-4 expression may well lead to a better understanding of the etiology of these diseases as well as potential therapeutic targets in their treatment.

## Materials and Methods

### Cell Lines and Plasmids

VDR145^+/+^ and VDRK240^−/−^ cells were a kind gift from Dr. JoEllen Welsh. They are maintained in DMEM-F12 (1∶1) (Invitrogen Cat. #11330-032) supplemented with 5% fetal bovine serum. CaCo2 cells were obtained by the ATCC and maintained in DMEM (Invitrogen 11995-065) supplemented with 5% fetal bovine serum. VP16 β-catenin was generated as described [2]. Wild-type VDR and VDRE-luc plasmids were a kind gift from Dino Moras [65]. The CMV-p53 construct was a kind gift from Dr. Maria Laura Avantaggiati. The TCF7L2 plasmid as well as the TopFlash and FopFlash were a kind gift from Marc van de Wetering [66]. pcDNA3.1 is available from Invitrogen (cat. #V79020). For cloning of the TCF7L2 reporter constructs, genomic DNA was harvested from VDR145^+/+^ cells and used to amplify the mouse TCF7L2 promoter with the following primers: -2068-luc: 5′-GGA CTG TGA TTC TCA CCC G-3′ (forward), and 5′-CCC CAA AAA AAT ACT GCA AGA A-3′ (reverse) using Invitrogen's Platinum Taq PCR amplification kit (Cat #10966-018) and TA cloned into pCR8/GW/TOPO vector (Cat #K250020SC). Recombination with a Gateway-compliant pGL3 expression vector, a kind gift from Dr. Caroline Hurley's laboratory [67], was performed with LR-Clonase (Invitrogen Cat #11791-019). Candidate clones were sequenced with RV3 (5′-CTA GCA AAA TAG GCT GTC C-3′) and GLprimer2 (5′- CTT TAT GTT TTT GGC GTC TTC C-3′) primers. The −1037 construct was generated with the following primers: (XhoI site added) 5′-CTC GAG GAG AGG TAA GCC TTC TTT TGG AC-3′ (forward); (HindIII site added) 5′-AAG CTT CAG CAG CAA TTT TGG AAG AAA AAT GA-3′ (reverse). Both amplicon and pGL3-Luc-Promoter (Promega Cat. #E1761) was digested with HindIII and XhoI (digestion with these two enzymes removes the SV40 promoter, making this plasmid analogous to pGL3-Luc-Basic) and cloned into these sites. All plasmids are used at .5 µg/µL.

### Western Blots

Caco-2 and VDRK240^−/−^ cells were seeded to ∼70% confluency and transfected with the indicated amounts of VDR, pcDNA3.1, or siRNA against VDR (Dharmacon Cat #LQ-003448-00) or a non-targeting siRNA (Dharmacon Cat #D-001810-10) using Lipofectamine 2000 (Invitrogen, Cat. #11668019) according to the manufacturer's recommendations. Cells were harvested in ice-cold RIPA buffer (50 mM Tris-HCl, pH 8.0, 150 mM NaCl, 1% NP-40, .5% sodium deoxycholate, 1 mM EDTA, .1% SDS,) supplemented with protease inhibitor cocktail (Roche Cat #11836153001, plus 1 mM sodium vanadate, and 50 mM sodium fluoride) with shaking for 15 minutes. After cell scraping lysates were drawn three times through an 18 Gauge needle and the insoluble fraction was removed by centrifugation at 21,000 g for 10 minutes at 4°C. Proteins were quantified via Bradford Assay (BioRad Cat. #s 500-0113, 500-0114, 500-0115). 10 µg of protein were separated on 4–12% Bis-Tris gels (Invitrogen Cat. #NP0336) with MES buffer (Invitrogen Cat. #NP0002) and transferred to Protran nitrocellulose (Whatman #10401396). Blots were blocked for at least an hour in 5% non-fat milk solution made up in PBS plus .1% Tween 20 (PBST), and then incubated overnight at 4°C with 1∶500 dilution of the following antibodies: TCF-4 (Cell Signaling Cat #2566, Cell Signaling Cat #2569, Upstate Cat #05-511), LEF-1 (Cell Sigalning Cat #2230), TCF-3 (Cell Signaling Cat #2883), TCF-1 (Cell Signaling Cat #2203VDR (Santa Cruz Cat. #sc-13133, BioMeda V4045). GAPDH control blots were incubated in 1∶10,000 dilution (Fitzgerald Cat. #10R-G109A) for 1 hour at room temperature. Blots were washed at least three times for 5 minutes each in PBST, followed by incubation with affinity-purified, peroxidase-labeled IgG secondary antibody (goat-anti-rabbit: KPL, Cat. #074-1506; goat-anti-mouse: KPL, Cat. #074-1806) diluted in 5% non-fat milk in PBST. Blots were exposed via chemiluminescence (GE Healthcare #RPN2106, Millipore Immobilon Cat #WBKLS0050).

### GST Pulldown

GST fusion proteins were generated and attached to GST beads as described [3]. 500 µg of protein from whole cell lysates of VDR145^+/+^ and VDRK240^−/−^ cells (harvested as described in Western Blot) were incubated with 20 uL of bead-bound GST-β-catenin, GST-dTCF-β-Cat, or beads alone for 4 hours at 4°C. Beads were collected by 1-minute centrifugation at 3000 rpm. Beads were washed three times with 500 µL of cold RIPA buffer. Proteins were eluted in 30 µL of sample buffer by heating at 92°C for 3 minutes. Proteins were separated on a polyacrylamide gel and processed as described in Western Blot.

### Luciferase Assays

#### OT/OF in VDRK240^−/−^ and VDR145^+/+^ cells

100,000 cells were seeded in 12-well plates. 150 ng of reporter, 40 ng of *Renilla* and 100 ng of VP16-β-catenin, TCF7L2, or both, were transfected into the cells via Lipofectamine PLUS (Invitrogen Cat. #10964-013) according to the manufacturer's protocol. 48 hours after transfection, proteins were collected with 300 µL Passive Lysis Buffer.

#### All other luciferase assays

CaCo2 and VDRK240^−/−^ cells were seeded in a 24-well plate to 70% confluency and allowed to adhere overnight. Cells were transfected using Lipofectamine 2000 at the recommended dilution. Cells were transfected with a total of .8 µg of DNA per well (CaCo2: .2 µg Reporter, .02 µg TK-*Renilla*, .6 µg VDR, pcDNA3.1, or ratio combination of both, or p53; VDRK240^−/−^: .4 µg Reporter, .04 µg TK-*Renilla*, .4 µg VDR, pcDNA3.1 or ratio combination of both, or p53). For experiments with mutated -2068-luc, “low” VDR is .2 µg and “high” VDR is .4 µg. Lipid complexes were removed after four hours and replaced with complete media. Cells were treated 24 hours after transfection with 10^−7^ M 1,25(OH)_2_D_3_ (Sigma Cat. #D1530) or an equal volume of ethanol (EtOH), the vehicle control in DMEM∶F12 plus 5% charcoal stripped fetal calf serum. Lysates were harvested by incubating with 70 µL Passive Lysis Buffer at room temperature for 15 minutes. Lysates were analyzed LARII and Stop & Glo. All reagents are components of the Dual Luciferase Kit (Promega Cat. #E1960). Results were normalized for transfection efficiency by dividing Luciferase readout by *Renilla* readout. Background reporter activity was accounted for by subtracting FopFlash from and empty-vector control from TCF7L2 reporter readouts. Data was plotted with GraphPad, Prism, and subjected to two-way ANOVA or t-tests to test for significant differences among samples.

### Quantitative PCR

CaCo2 and VDRK240^−/−^ cells were seeded in six-well plates to a confluency of 70–80%.

#### Exogenous VDR experiment

Cells were transfected with a total of 4 ug of DNA (VDR, pcDNA3.1 or a mixture thereof). 24 hours after transfection, cells were treated for an additional 24 hours with 10^−7^ M 1,25(OH)_2_D_3_. Total RNA was harvested using .5 mL of Trizol reagent (Invitrogen Cat #15596-018) followed by shaking with 100 µL of chloroform and 20 minutes at 21,000×g and 4°C. RNA was precipitated by adding an equal volume (300 µL) of 100% EtOH to the aqueous phase. RNA was captured, cleaned and eluted using RNeasy Kit (Qiagen Cat #74106). Total RNA was diluted to uniform concentration. RNA was subjected to reverse transcription PCR with Applied Biosystems TaqMan Reverse Transcription Reagents Kit (Cat #N8080234). Subsequent cDNA was used in a quantitative amplification reaction using Applied Biosystems TaqMan Universal Master Mix (Cat #4304437) with the following primer/probes: human CYP24A1 (Hs00167999_m1), human TCF7L2 (Hs01009053_m1), human GAPDH (Hs99999905_m1), mouse TCF7L2 (Mm01261075_m1), mouse β-actin (Mm01205647_g1). Data were normalized to the housekeeping gene (GAPDH or β-actin) and fold change was plotted as an average of at least three biological replicates.

#### Time course experiment

Cells were seeded and allowed to adhere for 24 hours. Complete media was replaced with media containing 5% charcoal-stripped FCS. Cells were treated 48-, 24-, 8-, 4-, and 2-hours before simultaneous harvest and RNA was collected and processed for qPCR as described above.

#### Cycloheximide experiments

CaCo2 cells were seeded at 70% confluency. CHX was dissolved in 100% EtOH at a final stock concentration of 25 mg/mL immediately before use. DMEM supplemented with 5% charcoal-stripped fetal calf serum was spiked with CHX (or an equal volume of 100% EtOH) to a final concentration of 200 µg/mL. From this, serial dilutions were made down to 12.5 µg/mL. Cells were pre-treated for 30 minutes with different concentrations of CHX and then with 10^−7^ M 1,25(OH)_2_D_3_ or EtOH was added to the CHX-containing media for 24 hours. RNA was harvested and subjected to qPCR as described but also using DKK-4 qPCR primers (Applied Biosystems Cat # Hs00205290_m1).

### Site-Directed Mutagenesis

Site-directed mutagenesis was performed using Stratagene's QuikChange II kit (Cat #200523-5). Primers were designed to mutate the two central nucleotides of each hexamer (bold) to two thymidine residues (underlined). The reverse compliment of each primer was also used in the PCR-based reaction. The sequences of the forward primers are as follows:

- 180VDRE: ttcccctcccctc**ctttct**cttt**tcttct**ccccaggagag;
- 1156VDRE: ggataaaacgccttct**tgttat**att**tattct**agtgggactttacattgaatg;
- 1505VDRE: cctaataaccaacaa**acttcc**atc**tcttct**cccagggctgcccacc

### Chromatin Immunoprecipitation

CaCo2 cells were seeded to 80% confluency in 15 cm dishes and allowed to adhere overnight. Cells were treated for four hours with 10^−7^ M 1,25(OH)_2_D_3_. Proteins and nucleic acids were crosslinked by adding formaldehyde to a final concentration of 1% and incubated for 15 minutes at 37°C. Crosslinking was stopped by adding .125 M glycine (in PBS with protease inhibitor tabs, Roche Cat #04693116001) for five minutes. Cells were washed twice with cold PBS plus protease inhibitor tabs, and scraped into 2 mL PBS. Cells were pelleted and re-suspended in 200 µL of SDS lysis buffer (1% SDS, 10 mM EDTA, 50 mM Tris-HCl pH 8.1). Cells were sonicated thrice at 15 seconds pulse-45 seconds ice at power level three on a Microson Ultrasonic Cell Disruptor (Model XL2007). Volumes were brought up to 2 mL by adding 1.8 mL of ChIP Dilution Buffer (.01% SDS, 1.1% Triton X 100, 1.2 mM EDTA, 167 mM NaCl, 16.7 Tris-HCl, pH 8.1) plus protease inhibitors. Protein concentration was assayed via Bradford Assay as described in the Western Blot section. 300 µg of protein was pre-cleared for 2 hours at 4°C with rotation using 50 µL salmon sperm DNA/BSA/protein A agarose beads (Millipore Cat #16–157) plus rabbit pre-immune serum (Thermo Scientific Cat #31884). Beads were removed by centrifugation for 5 minutes at 500×g. Input controls were collected at this time. 1 mg of the following antibodies was added to each pre-cleared sample and rotated overnight at 4°C: rabbit-anti-VDR (Santa Cruz Cat. #sc-1008), rabbit IgG (control) (Santa Cruz Cat. #sc-2027), rabbit-anti-RXR (Santa Cruz Cat #sc-553). Antibodies were pulled down by the addition of 50 µL protein A beads and rotation for 2 hours at 4°C. Bead/antibody/protein/DNA complexes were pelleted at 500×g for 2 minutes and washed by five-minute rotation with 1 mL of the following wash buffers: once with low salt buffer (.1% SDS, 1% Triton X 100, 2 mM EDTA, 20 mM Tris-HCl pH 8.1, 150 mM NaCl); once with high salt buffer (1% SDS, 1% Triton X 100, 2 mM EDTA, 20 mM Tris-HCl pH 8.1, 500 mM NaCl); once with LiCl buffer (.25 M LiCl, 1% NP40, 1% deoxycholate, 1 mM EDTA, 10 mM Tris-HCl pH 8.1), twice with TE buffer (10 mM Tris-HCl pH 8.1, 1 mM EDTA). Proteins were eluted with 2×15 minute rotations in 100 µL of 1% SDS, 0.1 M NaHCO_3_. Crosslinking was reversed by incubation of eluates with NaCl (final concentration .2 M) at 65°C overnight. Proteins were subsequently digested by addition of proteinase K (final concentration is 40 µg/mL) (Amresco Cat. #E195-5 mL) for 1 hour at 45°C. DNA was purified using Qiagen PCR Purification Module (Cat #28106). Eluates were used as templates in the amplification of regions of the human CYP24A1 and TCF7L2 promoters containing the putative VDREs with primers as listed:

hTCF7L2 VDRE +88F, 5′-GGA GGA GCT GTT TTG ATT CG-3′
hTCF7L2 VDRE +88R, 5′-AAG ACC CCC AAG CAG AAA AA-3′
hTCF7L2 VDRE −162F, 5′-TTT TTC TGC TTG GGG GTC TT-3′
hTCF7L2 VDRE −162R, 5′-TCC TTG GGA ACG AGA GAA AA-3′
hTCF7L2 VDRE −685F, 5′-GGG GCG GCT AAC AAT GAT-3′
hTCF7L2 VDRE −685R, 5′-ATT GTC TTT CTG AAA CCG CC -3′
hTCF7L2 VDRE −808F, 5′-GGC GGT TTC AGA AAG ACA AT-3′
hTCF7L2 VDRE −808R, 5′-CCC AAG TGG GCT TTC CTT-3′
hTCF7L2 VDRE −3656F, 5′-GCG AGA CTC CGT CTC AAA AA-3′
hTCF7L2 VDRE −3656R, 5′-CCT GGC TCA GGC TAA AGT CA-3′
hTCF7L2 VDRE −3827F, 5′-CCA AAT GGC AGA GAT TTG AA-3′
hTCF7L2 VDRE −3827R, 5′-TGC CTT CCT CCC AAA ATA TG-3′
hCYP24 ChIP F: 5′-CGA AGC ACA CCC GGT GAA CT-3′
hCYP24 ChIP R: 5′-CCA ATG AGC ACG CAG AGG AG-3′

### Density Experiment

CaCo2 cells were seeded in 6-well plates at the following concentrations: Low density: 200,000 cells/well (20% confluent); Medium density: 500,000 cells/well (50% confluent); High density: 1,000,000 cells/well (70% confluent). Cells were allowed to adhere overnight and then treated for 24 hours with 10^−7^ M 1,25(OH)_2_D_3_. Total RNA was harvested and subjected to qPCR as described.

### Statistics

Student's t-test was used to determine significant differences between samples in the luciferase assays for OT (Figure 1C and 1D) and TopFlash activity (Figure 7) (between VDR145^+/+^ and VDRK240^−/−^ cells); mRNA for TCF7L2 of treated CaCo2 and VDRK240^−/−^ cells (Figure 3A and Supplementary Figure S1B) (between EtOH- and 1,25(OH)_2_D_3_-treated samples); the time-course of 1,25(OH)_2_D_3_ treatment of CaCo2 cells (Figure 3B) (between 0-hour time-point and each individual time-point thereafter); density experiments (Supplementary Figure S3) (between EtOH- and 1,25(OH)_2_D_3_-treated samples); Cycloheximide experiments (Figure 6) (between EtOH- and 1,25(OH)_2_D_3_-treated samples). One-tailed t-tests were used to analyze protein densitometry of CaCo2 cells treated with 1,25(OH)_2_D_3_ (Figure 2E). Two-way ANOVA was used to analyze luciferase data from luciferase assays for -1037-luc plasmid (Figure 4C, left panel) (between EtOH- and 1,25(OH)_2_D_3_-treated samples); -2068-luc plasmid (Figure 4C, right panel and Supplementary Figure S4A), and is mutated progeny (Figure 5B and Supplementary Figure S4B) for a trend in expression with increasing VDR.

## Supporting Information

Figure S1 VDRK240^−/−^ cells are unable to increase TCF-4 protein or TCF7L2 mRNA expression in response to VDR and 1,25(OH)_2_D_3_. (A) Western blot for VDRK240^−/−^ cells transfected for different amounts of time with different amounts of VDR, as indicated in the presence of full-serum (5% FBS). (B) VDRK240^−/−^ cells were transfected for 24 hours with different amounts of VDR and treated for a subsequent 24 hours with 10^−7^ M 1,25(OH)_2_D_3_ or EtOH, as indicated. TCF7L2 mRNA was assayed by qPCR. Error bars represent SEM. No statistically significant differences were detected.
(0.73 MB TIF)

Figure S2 Several colorectal cancer cell lines increase TCF-4 protein in response to 1,25(OH)_2_D_3_. Densitometry of three replicates of western blots as performed as in Figure 2C. Data were plotted relative to each cell-line EtOH-control. Upper and lower TCF-4 bands were measured and plotted independently. Error bars represent SEM.
(0.58 MB TIF)

Figure S3 Density plays a role in the regulation of TCF-4 and CYP24A1 in CaCo2 cells. CaCo2 cells were seeded at three densities: Low (20%-), medium (50%-) and high (70%-confluency) and treated for 24 hours with 10^−7^ M 1,25(OH)_2_D_3_. mRNAs were analyzed by qPCR and plotted relative to the low-density EtOH treated sample. Error bars represent SEM. Statistics represent student's t-test: *: p<.05.
(0.52 MB TIF)

Figure S4 VDRK240^−/−^ cells are able to regulate TCF7L2 reporter constructs in response to VDR and 1,25(OH)_2_D_3_. (A) VDRK240^−/−^ cells were transfected with -2068-luc, Renilla and different amounts of VDR for 24 hours and treated for a subsequent 24 hours with 10^−7^ M 1,25(OH)_2_D_3_ or EtOH, as indicated. Luciferase proteins were analyzed and data were plotted relative to 0 µg VDR/EtOH sample. Error bars represent SEM. Statistics are generated with two-way ANOVA: ***: p<.0001. (B) -2068-luc construct containing all three sets of half-site mutations (d1502/d1153/d177) was transfected into VDRK240^−/−^ cells and treated with ligand as described in Figure 4C with only 3 concentrations of VDR (low, medium and high). Error bars represent SEM. Statistics represent analysis using two-way ANOVA: *p<.05. RLU-Relative Light Units.
(0.63 MB TIF)

Figure S5 CYP24A1 induction by 1,25(OH)_2_D_3_ is reduced but never abolished in response to cycloheximide treatment. CaCo2 cells were pre-treated for 30 minutes with different concentrations of the protein synthesis inhibitor, Cycloheximide (CHX) before addition of 10^−7^ M 1,25(OH)_2_D_3_ or EtOH for 24 hours, as indicated. Analysis of mRNA abundance of CYP24A1 was assayed by qPCR.
(0.54 MB TIF)

Figure S6 No other members of the TCF/LEF family are up-regulated in response to 1,25(OH)_2_D_3_ in CaCo2 cells. CaCo2 cells were treated with 10^−7^ M 1,25(OH)_2_D_3_ (right lane) or EtOH (left lane) for 24 hours. Whole cell lysates were blotted for LEF-1, TCF-3 and TCF-1. GAPDH was assayed to demonstrate even loading of lanes. Densitometry was measured and plotted relative to the EtOH-control band.
(1.00 MB TIF)

Table S1 (0.08 MB DOC)
