# Supplementary figures and images for: Control of TCF-4 Expression by VDR and Vitamin D in the Mouse Mammary Gland and Colorectal Cancer Cell Lines
Source: PLoS One. 2009 Nov 17;4(11):e7872. doi: 10.1371/journal.pone.0007872 (PMC2774944; doi:10.1371/journal.pone.0007872)

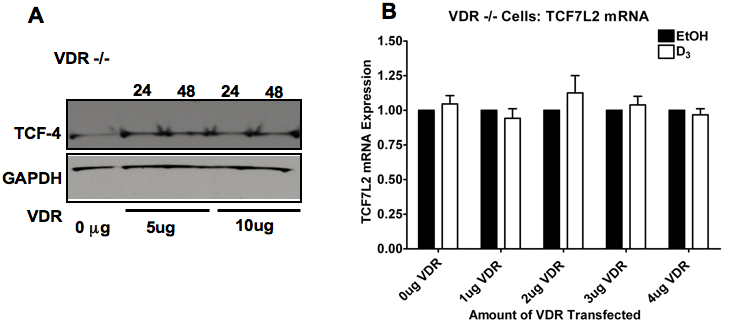

Supplement: Figure S1 — VDRK240−/− cells are unable to increase TCF-4 protein or TCF7L2 mRNA expression in response to VDR and 1,25(OH)2D3. (A) Western blot for VDRK240−/− cells transfected for different amounts of time with different amounts of VDR, as indicated in the presence of full-serum (5% FBS). (B) VDRK240−/− cells were transfected for 24 hours with different amounts of VDR and treated for a subsequent 24 hours with 10−7 M 1,25(OH)2D3 or EtOH, as indicated. TCF7L2 mRNA was assayed by qPCR. Error bars represent SEM. No statistically significant differences were detected. (0.73 MB TIF) [file pone.0007872.s001.tif]

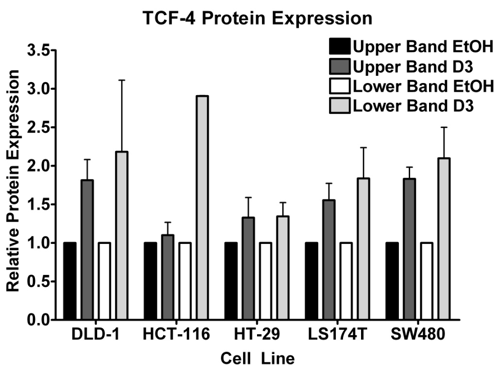

Supplement: Figure S2 — Several colorectal cancer cell lines increase TCF-4 protein in response to 1,25(OH)2D3. Densitometry of three replicates of western blots as performed as in Figure 2C. Data were plotted relative to each cell-line EtOH-control. Upper and lower TCF-4 bands were measured and plotted independently. Error bars represent SEM. (0.58 MB TIF) [file pone.0007872.s002.tif]

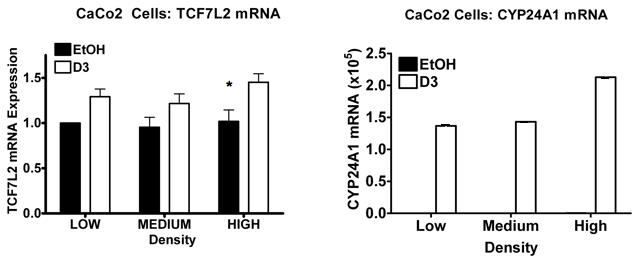

Supplement: Figure S3 — Density plays a role in the regulation of TCF-4 and CYP24A1 in CaCo2 cells. CaCo2 cells were seeded at three densities: Low (20%-), medium (50%-) and high (70%-confluency) and treated for 24 hours with 10−7 M 1,25(OH)2D3. mRNAs were analyzed by qPCR and plotted relative to the low-density EtOH treated sample. Error bars represent SEM. Statistics represent student's t-test: *: p<.05. (0.52 MB TIF) [file pone.0007872.s003.tif]

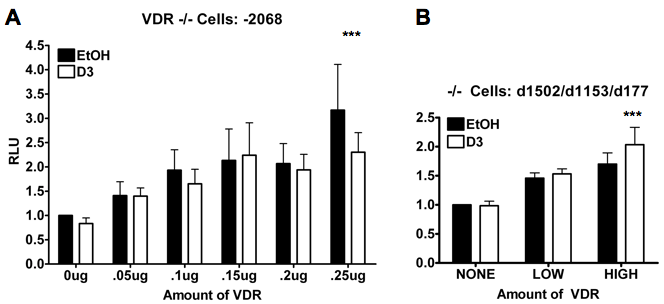

Supplement: Figure S4 — VDRK240−/− cells are able to regulate TCF7L2 reporter constructs in response to VDR and 1,25(OH)2D3. (A) VDRK240−/− cells were transfected with -2068-luc, Renilla and different amounts of VDR for 24 hours and treated for a subsequent 24 hours with 10−7 M 1,25(OH)2D3 or EtOH, as indicated. Luciferase proteins were analyzed and data were plotted relative to 0 µg VDR/EtOH sample. Error bars represent SEM. Statistics are generated with two-way ANOVA: ***: p<.0001. (B) -2068-luc construct containing all three sets of half-site mutations (d1502/d1153/d177) was transfected into VDRK240−/− cells and treated with ligand as described in Figure 4C with only 3 concentrations of VDR (low, medium and high). Error bars represent SEM. Statistics represent analysis using two-way ANOVA: *p<.05. RLU-Relative Light Units. (0.63 MB TIF) [file pone.0007872.s004.tif]

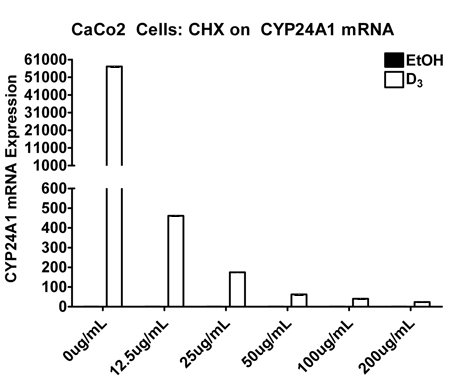

Supplement: Figure S5 — CYP24A1 induction by 1,25(OH)2D3 is reduced but never abolished in response to cycloheximide treatment. CaCo2 cells were pre-treated for 30 minutes with different concentrations of the protein synthesis inhibitor, Cycloheximide (CHX) before addition of 10−7 M 1,25(OH)2D3 or EtOH for 24 hours, as indicated. Analysis of mRNA abundance of CYP24A1 was assayed by qPCR. (0.54 MB TIF) [file pone.0007872.s005.tif]

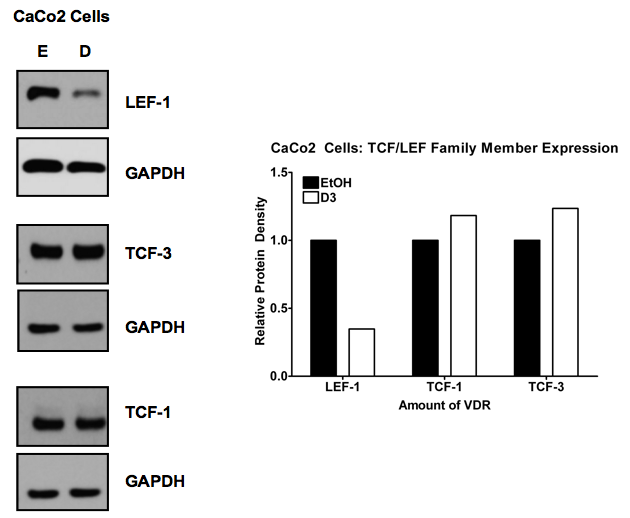

Supplement: Figure S6 — No other members of the TCF/LEF family are up-regulated in response to 1,25(OH)2D3 in CaCo2 cells. CaCo2 cells were treated with 10−7 M 1,25(OH)2D3 (right lane) or EtOH (left lane) for 24 hours. Whole cell lysates were blotted for LEF-1, TCF-3 and TCF-1. GAPDH was assayed to demonstrate even loading of lanes. Densitometry was measured and plotted relative to the EtOH-control band. (1.00 MB TIF) [file pone.0007872.s006.tif]
