# Supplementary material for: Control of TCF-4 Expression by VDR and Vitamin D in the Mouse Mammary Gland and Colorectal Cancer Cell Lines
Source: PLoS One. 2009 Nov 17;4(11):e7872. doi: 10.1371/journal.pone.0007872 (PMC2774944; doi:10.1371/journal.pone.0007872)
Supplement: Table S1 — (0.08 MB DOC) [file pone.0007872.s007.doc]

| **Mouse TCF7L2 Promoter** | | | | | |
| --- | --- | --- | --- | --- | --- |
| **Location** | **VDRE Type** | **5' or 3'** | **Sequence** | **Gene** | **Reference** |
| -177 | DR4 | 5' | TCTCCT | mouse Calbindin D28K | 32 |
| -177/-187 | DR4 | 3'/5' | AGGGGA | quail slow myosin heavy chain | 33 |
| -187 | DR4 | 3' | AGGGAG | human p21 | 34 |
| -1153 | DR3 | 5' | AGGTTA | rat parathyroid related peptide | 35 |
| -1153 |  | 3' | ATGCCA | mouse *c-fos* | 36 |
| -1502 | DR3 | 5' | AGTTGA | mouse RANKL | 37 |
| -1502 |  | 3' | GGGTGT | Rat calbindin D9K | 38 |
|  |  |  |  |  |  |
| **Human TCF7L2 Promoter** | | | | | |
| **Location** | **VDRE Type** | **5' or 3'** | **Sequence** | **Gene** | **Reference** |
| +85 | DR6 | 5' | TCTCCT | mouse Calbindin D28K | 32 |
| +85 |  | 3' | CGCCCT | rat, human, mouse CYP24A1 | 39 |
| -162 | DR4 | 5' | AGGGGA | quail slow myosin heavy chain | 33 |
| -162 |  | 3' | AGGGAG | human p21 | 34 |
| -685 | DR3 | 5' | GGGAGA | chicken integrin β3 | 40 |
| -685 |  | 3' | AGGGAG | human p21 | 34 |
| -808 | DR4 | 5' | GTGGGA | human growth hormone | 41 |
| -808 |  | 3' | AGGAGA | mouse Calbindin D28K | 32 |
| -3646 | DR4 | 5' | AGTTGA | mouse RANKL | 37 |
| -3646/-3656 | DR6 | 3'/5' | GGTCCA | mouse *c-fos* | 36 |
| -3656 |  | 3' | GAGGCA | chicken integrin β3 | 40 |
| -3827 | DR3 | 5' | GTGGGA | human growth hormone | 41 |
| -3827 |  | 3' | GGGAGA | chicken integrin β3 | 40 |
